# Overview: Report of a Scientific Working Group on Serious Adverse Events following Mectizan^®^ treatment of onchocerciasis in Loa loa endemic areas.

**DOI:** 10.1186/1475-2883-2-S1-S1

**Published:** 2003-10-24

**Authors:** Brian OL Duke

**Affiliations:** 1 River Blindness Foundation, 2 Hillside, Lancaster, LA1 1YH, United Kingdom

This report reviews information on Serious Adverse Events (SAE), mainly *Loa*-encephalopathy, following treatment with ivermectin (Mectizan^®^, Merck, Sharpe & Dohme) for control of onchocerciasis carried out by the African Programme for Onchocerciasis Control (APOC), in areas where heavy microfilarial infections with *Loa loa *are co-endemic with *Onchocerca volvulus *infections. It also endeavours to define the information and research needed to understand, prevent, and manage cases of *Loa*-encephalopathy.

In coping with the recent appearance of the risk of *Loa*-encephalopathy, the Mectizan^® ^Expert Committee and its secretariat, the Mectizan^® ^Donation Program (MDP), and the national onchocerciasis control programmes, involving APOC and associated Non-Governmental Development Organizations (NGDOs), have to assess the benefits of mass treatment in preventing the blindness and unpleasant skin manifestations of onchocerciasis in large numbers of infected persons, against the risk of inciting severe and sometimes fatal reactions of *Loa*-encephalopathy in a small proportion of treated persons living in areas of high *Loa *co-endemicity. In practical terms, this dilemma raises a number of problems and implies the need for much extra effort and costs in the field prior to, during, and shortly after community distribution of Mectizan^®^.

As Haselow *et al.*, [1] point out, the risk of *Loa*-encephalopathy demands better explanation of the benefit/risk ratio in those communities to be treated by APOC wherein *L. loa *and *O. volvulus *are co-endemic, and also more careful control of stocks of Mectizan^®^. It demands a reinforced health-education campaign in favour of taking Mectizan^®^. It will mean confining Mectizan^® ^treatment more rigidly to hyper- and meso-endemic onchocerciasis communities, which in turn implies more accurate Rapid Epidemiological Assessment (REA) to exclude hypo-endemic onchocerciasis communities. Where treatment is to go ahead, the population must be made aware, without inciting undue alarm, of the early signs of *Loa*-encephalopathy so as to facilitate rapid referral of such cases to hospital; and each potential referral hospital needs to have a team of at least four persons who are trained to deal with *Loa*-encephalopathy cases. In terms of practical field assessment, the models explored by Addiss *et al.*, [2] suggest that accurate REA identification of hyper- and meso-endemic onchocerciasis communities, coupled with a RAPLOA limit of 20% of persons with a history of *Loa *worms crossing the eyeball, is probably the currently best safety limit to apply.

Collections of data from past reports submitted to the MDP have been carefully analysed by Twum-Danso [3,4] who indicates that, among all 207 SAEs recorded by passive surveillance during 165,000,000 Mectizan^® ^treatments (1:800,000) carried out between 1989 and 2001, there were 65 (31·4%) cases of "probable" or "possible" *Loa*-encephalopathy temporally related to Mectizan^® ^ treatment. Of them:

i) 85 % were in males;

ii) 93% followed the first dose of ivermectin;

iii) 97% were from Cameroon;

iv) The mean age of the subjects was 39·9 years (Range 6 – 89)

v) Mean time to onset of symptoms was 1·7 days;

vi) Mean time to first medical attention was 2·0 days;

vii) Mean period of hospitalization (53 cases) was 27·5 days

Outcome: (34 cases):

i) 23·5% were fatal;

ii) 11·8% had partial residual neurological deficit;

iii) and 64·7% recovered fully

Mean concentrations of microfilariae (mfs) *Loa *in the peripheral blood (32 cases) were:

i) Pre-treatment – 164,250 mfs/ml

ii) Post-treatment (< 1 month) – 3,926 mfs/ml

iii) Post-treatment (< 5–6 months) – 7,800 mfs/ml

The clinical manifestations of *Loa*-encephalopathy are well described by Boussinesq *et al. *[5]. Hospital staff in areas at risk of *Loa*-encephalopathy need to be familiar especially with:

i) the neurological symptoms and signs, and haemorrhages of the palpebral conjunctiva (HPCs) and their time of onset;

ii) the vital importance of good-nursing of cases to prevent bed-sores and of re-hydration;

iii) the likely duration of the various stages of the disease; and

iv) the probable diagnostic concentrations of *Loa *mfs to be found in such cases at various intervals *post*-treatment.

All the above considerations will inevitably add considerable costs and effort to the activities of the MDP and APOC in the co-endemic *Loa *areas concerned.

To date, cases of sometimes fatal ivermectin-induced *Loa*-encephalopathy show a very remarkable distributional clumping in one particular Health Area (Lékié) in the Republic of Cameroon; but it should be remembered that similar *Loa*-encephalopathies have been reported following treatment of individual loiasis sufferers with diethylcarbamazine citrate (DEC), which is both a potent microfilaricide and a fairly effective macrofilaricide for *L. loa. *Again there was evidence of distributional clumping, this time in the Mayumbe Region of the Democratic Republic of the Congo (DRC), where 80–90 cases were recorded over 10 years in a single hospital [6]. This appears to be another potentially dangerous area that is only now about to be covered by the activities of APOC, and one that may well give rise to difficulties. This remarkable clumping of cases may eventually provide a clue to the causation of *Loa*-encephalopathy. On the other hand, more mundane aspects involving the different Health Areas, particularly in Cameroon, need also to be taken into account. The authorities in Health Areas that provide free hospital treatment for sufferers (*e.g. *Lékié) are likely to be made aware of many more cases of *Loa*-encephalopathy than those in which patients are expected to pay for their treatment and may thus prefer to remain hidden in their homes.

The Report emphasizes the importance of an experimental approach to determine

*(i) *the factors governing the remarkably uneven distribution of cases within the *Loa*-endemic area;

*(ii) *the practical means of detecting communities that are at high risk of *Loa*-encephalopathy;

*(iii) *the clinical symptoms and signs that characterise *Loa*-encephalopathy and the recommendations for its clinical management;

*(iv) *the means of allaying the fears of the inhabitants that they may suffer from *Loa*-encephalopathy; and

*(v) *the responsibilities of the Ministries of Health, APOC, the national/APOC/NGDO Mectizan^® ^ distribution teams and of individual physicians and supporting hospital staff in dealing with them.

The exact pathogenesis of *Loa*-encephalopathy is not presently understood, but the most probable primary factor involved is the high concentration of *Loa *microfilariae in the blood-stream. This problem could well be investigated in experimentally-infected monkeys, especially *Mandrillus leucophaeus *[7] and *M. sphinx*, both of which are good natural hosts of the nocturnally-periodic simian *Loa *and can also readily be infected with the diurnally-periodic, human *L. loa. *In the simian host, the spleen becomes enlarged and granulomatous as it accomplishes its function of destroying a large proportion of the circulating *Loa *microfilariae [8,9]. When the spleen is removed surgically, very high concentrations of microfilariae will develop in the peripheral blood (of the same order – up to 50,000/ml – as those seen pre-treatment in human *Loa*-encephalopathy patients), providing what is probably an excellent model for studying drug-induced *Loa*-encephalopathy. Furthermore, the diurnally-periodic human and the nocturnally-periodic simian parasites can be hybridised in the simian host [10].

Although the main *Chrysops *vectors of the human and simian parasites are different (day-biting at ground-level and attracted by wood-smoke, for *Chrysops silacea *and *C. dimidiata*, the main vectors of the human parasite; and night-biting at forest canopy-level for *C. langi *and *C. centurionis*, vectors of the simian parasite) [11], this raises the possibility that under forest-dwelling conditions humans may, over the course of time, become infected with simian or hybrid strains of *Loa *having larger microfilariae and possibly also different periodicities or other characteristics. It is noteworthy that some of the patients with *Loa*-encephalopathy following DEC treatment in the Mayumbe area of the DRC showed abnormal periodicities, some of which were predominantly nocturnal. It is also interesting that the Sanaga River, which flows through Cameroon, is a well-known faunal barrier that separates *M. leucophaeus *on the north-west bank from *M. sphinx *on the south-east bank. It is thus possible that different strains of human and simian *Loa *may exist and may have spread to, or hybridised in, the human population in different areas.

Not only may studies of *Loa *in monkeys (recently re-initiated in Cameroon by Drs. S. Wanji and P. Enyong) reveal the possible influence of strains or sub-species of *Loa *on the development of *Loa*-encephalopathy, but they should facilitate investigations into the biochemical, haematological and histopathological changes that occur in this condition, thus elucidating the mechanism, pathogenesis, prevention and treatment of *Loa*-encephalopathy in splenectomised monkeys with very high *Loa *mfs counts.

In the Report, careful consideration is also given by Edwards *et al.*, [12] and Mackenzie *et al.*, [13] to the possible effects of a putative gene mutation in humans (analogous to that found in collie dogs and collie cross-breeds) which renders its carriers fatally susceptible to the effects of ivermectin. An absence or functional deficiency in P-glycoprotein production (or its inhibition by alcohol or some other food-stuff) in the apical membrane of the brain capillary epithelial cells could result in dangerous penetration of ivermectin into the brain. Informed current opinion is that this putative alternative pathogenesis of *Loa*-encephalopathy is less plausible than the mfs-embolic hypothesis, but that the existence of any such putative gene deficiency in humans could be determined by experiment.

Another possible mechanism for *Loa*-encephalopathy is the release of *Wolbachia *organisms from dying adult worms but, as these endosymbionts are seldom found in *Loa *[14,15], this hypothesis is now considered somewhat unlikely.

Finally, the report suggests that the prevention of *Loa*-encephalopathy in humans could also be investigated directly as, for example, by studying the potentialities of using lower doses of ivermectin or by pre-treatment with albendazole designed to lower the basic concentrations of *Loa *microfilariae at a slower rate.
